# Schedule-Dependent Synergy Between the Histone Deacetylase Inhibitor Belinostat and the Dihydrofolate Reductase Inhibitor Pralatrexate in T-and B-cell Lymphoma Cells *in vitro*

**DOI:** 10.3389/fcell.2020.577215

**Published:** 2020-10-09

**Authors:** Godefridus J. Peters, Frank P. A. van Gemert, Ietje Kathmann, Guru Reddy, Saskia A. G. M. Cillessen, Gerrit Jansen

**Affiliations:** ^1^Department of Medical Oncology, Amsterdam UMC, VU University Medical Center Amsterdam, Amsterdam, Netherlands; ^2^Department of Biochemistry, Medical University of Gdańsk, Gdańsk, Poland; ^3^Spectrum Pharmaceuticals, Irvine, CA, United States; ^4^Department of Pathology, Amsterdam UMC, VU University Medical Center Amsterdam, Amsterdam, Netherlands; ^5^Amsterdam Rheumatology and Immunology Center, Amsterdam UMC, VU University Medical Center Amsterdam, Amsterdam, Netherlands

**Keywords:** antifolates, pralatrexate, histone deacetylase inhibitors, belinostat, peripheral T-cell lymphoma, diffuse B-cell lymphoma

## Abstract

Pralatrexate (Folotyn; PLX) and belinostat (Beleodaq; BLS) are registered for the treatment of patients with peripheral T-cell lymphoma (PTCL) and are being considered for other lymphomas. In this study we investigated whether BLS had the ability to potentiate the cytotoxicity of PLX. A panel of lymphoma cell lines was used for the combination studies: the B-cell SUDHL-4, SUDHL-5, HT, Jeko-1 and T-cell Karpas-299 and Hut-78. Uptake of PLX was mediated by the reduced folate carrier (RFC). PLX showed a 6-fold better RFC substrate affinity compared to methotrexate, and 2-fold better than levoleucovorin (l-LV). Sensitivity expressed as the concentration that resulted in 50% growth inhibition (IC50) after 72 hr exposure to PLX varied from 2.8 to 20 nM and for BLS from 72 to 233 nM, independent of the background of the cell lines. The interaction between BLS and PLX was studied using the median-drug effect analysis. At a fixed molar ratio between the drugs based on the IC50 concentration the average combination index (CI) for all cell lines showed additivity (CI: around 1.0). In three selected cell lines (SUDHL-4, SUDHL-5, and HT) sequential exposure (24 h pretreatment with BLS, followed by 48 h to PLX + BLS), did not improve interaction (CI: 0.9–1.4). As an alternative approach a non-fixed ratio was used by exposing SUDHL-4, SUDHL-5, and HT cells to IC25 concentrations of either BLS or PLX in combination with the other drug. Exposure to IC25 of PLX did not decrease the IC50 for BLS (CI from 0.6–1.2), but exposure to IC25 of BLS markedly increased PLX sensitivity (low CIs from 0.40 to 0.66). Mechanistic studies focused on induction of apoptosis, and showed cleavage of predominantly caspase-9 in HT and SUDHL-4 cells for both drugs at their IC50s, being similar in the combination setting. Moreover, at these concentrations, the drugs were shown to confer an S-phase arrest. In conclusion, the combination of PLX and BLS showed additivity in various lymphoma cell lines, with a schedule-dependent synergism in B-cell lymphoma. Based on these data, proficient inhibition of HDAC activity by BLS holds promise in sensitization of tumor cells to PLX.

## Introduction

Peripheral T-cell Lymphoma (PTCL) accounts for over 6,000 to 9,000 cases in the United States annually and worldwide this type of cancer represents 10 to 15% of all non-Hodgkin’s lymphoma (Rudiger et al., 2002; Laribi et al., 2018). PTCL is considered to be an aggressive form of T-cell malignancies with poor prognosis. The overall response rate for PTCL patients treated with CHOP (Cyclophosphamide-Hydroxydaunorubicin-Oncovin-Prednisone) chemotherapy is 30–60% and the 5-year survival rate is estimated around 15–20%. Despite poor outcome, CHOP and CHOP based chemotherapy programs form first-line treatment for PTCL (Gooptu et al., 2015; Laribi et al., 2018).

In 2009 the FDA approved the antifolate Pralatrexate (PLX) for the treatment of patients with relapsed or refractory PTCL (Shimanovsky and Dasanu, 2013). PLX is a folate analog and a potent dihydrofolate reductase (DHFR) inhibitor, designed to accumulate in cancer cells via the reduced folate carrier (RFC) and retained via efficient polyglutamylation (Gonen and Assaraf, 2012). Although PLX has been tested in solid tumors (Azzoli et al., 2007), it was ineffective. Compared with conventional antifolates, such as the classical methotrexate, PLX may be more tumor specific due to greater affinity for RFC (Wang et al., 2003) and polyglutamylation efficiency by folylpolyglutamate synthetase (O’Connor et al., 2011; Visentin et al., 2014). These two properties improve (tumor specific) cellular uptake and inhibit potential efflux following uptake. PLX was designed to be a better substrate for RFC, but other transporters facilitating the uptake of folates and antifolates could be involved (Gonen and Assaraf, 2012). These include the folate-receptor α or β (FRα,β) (Westerhof et al., 1995a, b; van der Heijden et al., 2009; Zhao et al., 2009; Frigerio et al., 2019), representing a high affinity low capacity transporter, and the proton-coupled folate transporter (PCFT), which is a high capacity transporter with an acidic pH optimum of about 5.5 (Zhao et al., 2008; Matherly et al., 2018). PLX competitively inhibits DHFR (DeGraw et al., 1993), an enzyme that converts dihydrofolate (DHF) into tetrahydrofolate (THF). Inhibition of DHFR leads to a reduction in the THF pool. THF is an essential cofactor required for the synthesis of purines and thymidine monophosphate (TMP). Therefore, inhibition of DHFR by PLX results in depletion of purines and dTMP, leading to an imbalance of deoxynucleotides, with a depletion of deoxythymidine triphosphate (dTTP) and an increase in deoxyuridine triphosphate (dUTP) resulting in DNA strand breaks and in inhibition of DNA synthesis (Marchi et al., 2010). Based on these properties, PLX has been tested in several other hematological and solid tumors as a single agent and in various combinations (Marchi et al., 2010, 2013; Serova et al., 2011; Dovzhanskiy et al., 2012). Antifolates can also be considered as epigenetic drugs since they affect cellular methylation reactions, due to inhibition of one-carbon metabolism (Frigerio et al., 2019).

Belinostat (BLS) is a hydroxamic acid-based pan-histone deacetylase (HDAC) inhibitor that inhibits all of the zinc-dependent HDAC enzymes, with high affinity for the Class I, II and IV isozymes (Eckschlager et al., 2017). HDAC inhibition results in an alteration in the degree of histone and non-histone protein acetylation, which in turn affects transcription of genes essential in cellular proliferation, cell cycle and DNA repair (Verza et al., 2020). Hence BLS is an epigenetic drug (Yeon et al., 2020) and is approved for the treatment of PTCL, but may have some activity against B-cell lymphomas as well (Tula-Sanchez et al., 2013). HDACs are a group of enzymes responsible for the deacetylation of histones, the core nucleosomal protein. Deacetylation of histones enables the DNA to wrap itself more tightly around the histone (Eckschlager et al., 2017; Verza et al., 2020). Inhibition of this event will keep histones in an acetylated state in which the DNA will be more accessible for transcription (Vandermeers et al., 2013). The acetylated state allows transcription of proteins involved in cell cycle arrest and other tumor suppressor genes (Ramalingam et al., 2009; Eckschlager et al., 2017), enabling an increase in cell death. HDAC inhibition leads to a decrease in the activity of DNA repair enzymes, preventing DNA damage, caused by DNA damaging drugs, being repaired (Verza et al., 2020). We reasoned that the epigenetic inhibition of HDAC would lead to decreased DNA repair, so that DNA damage caused by PLX, would not be repaired.

Therefore, both the efficacy of BLS and PLX for relapsed PTCL patients, and a potential synergistic/additive interaction of these drugs, encouraged us to investigate their combination. Although PLX and BLS have different intracellular targets, we hypothesize that PLX and BLS will interact at least additive and possibly synergistically, inducing more apoptosis than would be expected on their separate effects. We investigated the interaction of these two drugs in a panel of B- and T-cell lymphomas and determined whether the effect of the combination was mediated by an increased cell death.

## Materials and Methods

### Materials

RPMI-1640 medium, Fetal Bovine Serum, penicillin/streptomycin (100 μg/mL) and Hank’s Balanced Salt Solution (HBSS) were purchased from Lonza (Basel, Switzerland). Cell culture flasks were purchased from Greiner Bio one. l-Leucovorin (l-LV, Fusilev^®^), PLX and BLS were a gift from Spectrum (Irvine, CA, United States), Pemetrexed (PMX) was a gift from Eli Lilly and Company (Indianapolis, IN, United States). Folic acid was from Sigma Chemical Co (St. Louis, MO, United States). The drugs were first diluted in dimethyl sulfoxide, and then in medium before use. Radioactive compounds, [3′,5′,7,9-^3^H(N)]-(6S)-Leucovorin (25 Ci/mmol) and, [3′,5′,7,9-^3^H]-folic acid, diammonium salt (21.0 Ci/mmol) were obtained from Moravek Biochemicals (Brea, CA, United States).

### Cell Culture

Six lymphoma cell lines were used (Cillessen et al., 2008) in comparison with the T-cell leukemic cell line CCRF-CEM (Mauritz et al., 2002). The B-cell lymphoma cell lines were: SUDHL-4, SUDHL-5, HT, and Jeko-1. The T-cell lymphoma cell lines were Karpas-299 and Hut-78. The lymphoma cell lines were cultured at 37°C in 5% CO_2_ and 100% humidity in RPMI-1640 supplemented with 10% fetal bovine serum in 75 cm^2^flasks.

### Folate Receptor Binding and Transport Studies

In order to determine to which extent PLX was a substrate for either the folate receptors, RFC or PCFT we used competition experiments in cell lines overexpressing either transporter (Westerhof et al., 1995a, b; Jansen et al., 1997; Ifergan et al., 2008; Lasry et al., 2008; van der Heijden et al., 2009). For FRα and FRβ, we used an intact cell binding assay for competitive binding with KB cells and CHO-FRβ cells, respectively. Relative affinities were defined as the inverse ratio of compound to displace 50% of radio-active folic acid from FR-positive cells, with the relative affinity of folic acid set at 1. For RFC we used a competition assay with 5 μM [^3^H]-l-LV in CCRF-CEM/7A cells (RFC+++) for 2 min at 37°C. CEM-7A cells overexpress the RFC 30-fold over wild-type CCRF-CEM cells (Jansen et al., 1990, 1997). Relative affinities are expressed as the concentration of unlabeled drug necessary to inhibit [^3^H]-l-LV uptake by 50%.

The affinity for PCFT of PLX was assessed in competition with 2.5 μM ^3^H-l-LV at pH 5.5 in CHO-cells lacking RFC and transfected with PCFT. Pemetrexed (PMX) served as reference drug. The accumulation of 2.5 μM [^3^H]-l-LV was performed at 37°C; essentially as described for the RFC assay, but the incubation time was 3 min and was performed at pH 5.5, the optimal pH for PCFT, with increasing amounts of unlabeled drug. Also for PCFT relative affinities were expressed as the concentration of unlabeled drug necessary to inhibit [^3^H]-LV influx.

### Cell Growth Inhibition Experiments

Growth inhibition of the suspension cell lines (lymphoma and CEM) was routinely determined with the (3-[4,5-dimethylthiazol-2-yl]-2,5 diphenyl tetrazolium (MTT) assay (Keepers et al., 1991), however, some cell lines (the HT and SUDHL-4 cell lines) did not grow optimally in 96-well plates and therefore growth inhibition was assessed using cell counting before and after exposure using a cell counter. Cells were plated in 96-wells flat bottom plates, at 3,000 or 6,000 cells per well in culture medium (75 μL/well) or in 24-wells plates (60,000 cells/well) for the counting assays for which a Coulter Counter was used. Cells were treated immediately with a drug dilution series in cell culture medium (75 μL/well). After an incubation period of approximately 72 h, growth in the 96-wells plates was assessed by adding 15 μL MTT solution (stock solution 5 mg/ml in PBS, final concentration 0.5 mg/ml in culture medium) to each well. After incubation for 3 h in the 5% CO_2_ incubator with 100% humidity at 37°C, 150 μL acidified 2-propanol was added to the plates to dissolve the formazan crystals. The yellow-colored MTT stock solution is taken up and reduced to a purple formazan in living cells, which directly correlates to the amount of viable cells. As final step, the plates were measured at 540 (or 492) nm by the SPECTRAfluor spectrophotometer. Drug sensitivity was evaluated by determination of the IC50 value, which is the drug concentration resulting in 50% growth inhibition.

Drug interaction in combination experiments was evaluated using the median-drug effect analysis using CalcuSyn software (Bijnsdorp et al., 2011). In order to evaluate combination indexes [CI] from different experiments, the CI values at Fraction Affected (FA) values >0.5 (preferably around 0.5, 0.75, and 0.9) were averaged for each specific experiment. The means of the separate experiments were subsequently averaged so that a mean ± Standard Error of the Mean (SEM) was calculated from three or more separate experiments. Experimental conditions with a growth inhibition of <50% (equivalent to an FA < 0.5) are generally considered as clinically less relevant, since it only represents a minor level of growth inhibition (or in clinical terms a slightly less rapid growth than untreated tumors). Drug combinations were designed based on the standard CalcuSyn approach in which a series of drug concentrations were combined in a fixed molar ratio, based on the IC50 of each drug. A variation of this approach was a sequential scheduling in which cells were exposed for 72 h to BLS, while in the combination PLX was added after 24 h and cells were exposed from 24 to 72 h. In the non-fixed ratio approach one drug was used at the IC25 (concentration that results in 25% growth inhibition) and the other drug was added in a concentration range.

### Western Blotting

Western blotting was performed, essentially as described earlier (Bijnsdorp et al., 2010; De Wilt et al., 2012). In short, cell pellets of 5 × 10^6^ cells were harvested after exposure to the drugs for 24, 48, or 72 h. Cells were snap-frozen in liquid nitrogen and stored at −80°C. Before blotting the protein content was determined using the Bicinchoninic based protein assay, as described previously (Peters et al., 2014). The cell pellets were dissolved in 200 μL lysis buffer containing a protease inhibitor cocktail, sonicated and loaded on precast gels (Bio-Rad^TM^), electrophoresed for 45 min at 150 V, transferred to a polyvinylidene fluoride (PVDF) membrane and incubated with a suitable antibody against a cleaved caspase (mouse anti-caspase 8, #9748 (Cell signaling, Danvers, MA, United States; dilution 1:1000) and rabbit-anti-caspase 9, # 9501 [Cell Signaling, Danvers, MA, United States; dilution 1:1000), followed by a second antibody (anti-mouse or anti-rabbit (Sigma-Aldrich Chemicals, Zwijndrecht, Netherlands; dilution 1:10,000)], for Enhanced chemiluminescence. β-actin (antibody from Sigma-Aldrich, Zwijndrecht, Netherlands; dilution 1:10,000) was used as control for equal protein loading on the gels.

### Estimation of Cell Cycle Distribution and Cell Death Using FACS (Fluorescence Activated Cell Sorting)

Propidium iodide staining (DNA staining) was used to determine cell cycle distribution and the extent of cell death. Cells (120,000 per well in a 24-wells plate) were exposed to PLX or BLS or the combination for 24, 48, or 72 h. Cells were harvested, suspended in 1.0 ml phosphate buffered saline/1% Bovine serum albumin and fixed with 70% ethanol. After centrifugation the pellet was resuspended in hypotonic propidium iodide (0.1 mg/ml), Triton-X-100 (0.1%), sodium citrate (1 mg/ml) and RNAse A (0.5 mg/ml) (final concentrations in saline) and stored in dark on ice for 30 min (De Wilt et al., 2012). DNA content of the cells to determine cell cycle effects and cell kill was analyzed by a FACScalibur (Becton Dickinson Immunocytometry Systems, San Jose, CA, United States) with an acquisition of 10,000 events as described previously (De Wilt et al., 2012).

### Statistical Analysis

Experiments were performed at least in triplicate. Data were expressed as mean ± SEM and analyzed by a *t*-test. Level of significance is *p* = 0.05, if not otherwise stated.

## Results

### Substrate Specificity of PLX for Folate Transporters

**FIGURE 1:**
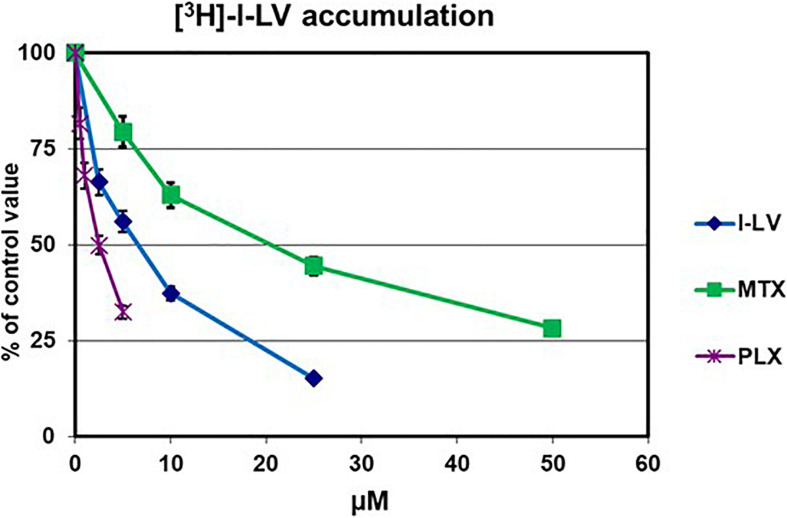
Evaluation of substrate specificity of PLX for the RFC. Transport was determined by evaluation of the uptake of 5 μM [^3^H]-l-LV for 2 min in CCRF-CEM-7A cells, which overexpress RFC. Specificity for the RFC was determined by the extent of inhibition by increasing concentrations of l-LV, PLX and MTX. Values are from one representative experiment in triplicate; performed three times. Error bars represent SEM, when not visible, they are within the size of the marker.

Upon development of PLX, it was anticipated that it would be an excellent substrate for the RFC and be suitable for treatment of malignancies with a high RFC expression (Tonner et al., 2006; Marchi et al., 2013). In order to exclude the contribution of other transporters in our assays we also determined the substrate specificity of PLX for other folate receptors and transporters. PLX was an excellent substrate for the RFC, even better than methotrexate (*P* < 0.001), which is considered to be one of the best substrates (Figure 1 and Table 1).). In contrast, PLX was a poor substrate for FRα (relative affinity of 0.0035 compared to 1 for folic acid), and even lower for FRβ (<0.001 compared to 1 for folic acid). PLX was also a very poor substrate for PCFT, both at the optimal pH of 5.5, and at the physiological pH of 7.4; 15 μM PLX were needed to displace 2.5 μM l-LV in contrast to 0.4 μM pemetrexed or 4 μM l-LV (*p* < 0.001 and *p* < 0.05, respectively) (Table 1). Therefore it can be concluded that PLX is primarily taken up by the RFC.

**TABLE 1:** Substrate specificity of PLX for RFC, PCFT and FRα.

	RFC	PCFT	FR-α
PLX	2.8 ± 0.1	14.8 ± 2.7	0.0035 ± 0.0005
l-LV	5.5 ± 0.7	4.0 ± 0.1	0.073 ± 0.0010
MTX	12.8 ± 1.3		
PMX		0.4 ± 0.1	
FA			1

*The units are defined as follows: For RFC: Concentration (μM) required to inhibit RFC mediated uptake of [^3^H]-l-LV (5 μM) in CCRF-CEM/7A cells via RFC by 50%. For PCFT: Concentration (μM) required to inhibit [^3^H]-l-LV (2.5 (μM) uptake by 50% in CHO-C5-PCFT cells at pH 5.5. The uptake of l-LV at pH 7.4. was 11% of that at pH 5.5. For FR-(α: the inverse ratio of compound to displace 50% of [^3^H] folic acid (FA) from FR-positive cells, with the relative affinity of FA set at 1 in each experiment. Values are means ± SEM of three separate experiments.*

### Sensitivity of Lymphoma Cell Lines to PLX and BLS

Since PLX and BLS are registered for the treatment of PTCL (O’Connor et al., 2011; Gooptu et al., 2015), we investigated the sensitivity of several other lymphoma cell lines, including B-cell, to these drugs, in comparison to the CEM cells used for transport studies (Table 2). We also characterized some of the cell lines for protection by l-LV, since LV (either as l-LV or as the racemic dl-LV) is often used to protect against MTX side effects. PLX is a very effective drug against the tested lymphoma cell lines, with IC50 values in the low nM range for Hut-29 and the SU cell lines and CEM, although Jeko and Karpass-299 cells were less sensitive. Cytotoxicity of the antifolate could be reversed very efficiently by 5 μM l-LV 201–6182-fold for the lymphoma cell lines, and 2965-fold for the CEM cells. Interestingly, the variation in sensitivity to BLS was less and varied from 72–233 nM. l-LV did not affect sensitivity to BLS (data not shown). These lymphoma cells seem well suited for combination studies of these two drugs.

**TABLE 2:** Sensitivity of CEM cells and lymphoma cells to PLX and BLS.

Cell line	Origin	PLX	PLX + l-LV	BLS
		*IC*_50_	*IC*_50_	*IC*_50_
CCRF-CEM	T-cell leukemia	2.6 ± 0.4	7775 ± 3234	95.8 ± 16.1
HT	B-cell lymphoma	7.4 ± 0.5	45750 ± 4921	223 ± 23
SUDHL 4	B-cell lymphoma	5.6 ± 0.4	1462 ± 71	72.3 ± 5.0
SUDHL 5	B-cell lymphoma	5.5 ± 0.9	1109 ± 351	112 ± 15
Jeko-1	B-cell lymphoma	18.7 ± 4.2		103 ± 11
Karpas-299	T-cell lymphoma	24.7 ± 5.3		203 ± 21
Hut-29	T-cell lymphoma	2.8 ± 0.5		97 ± 12

*IC_50_ values (in nM) are depicted as means ± SEM of 3-5 separate experiments. Cells were exposed to the drugs for 72 h. l-LV concentration: 5 μM.*

### Synergism Between PLX and BLS

**FIGURE 2:**
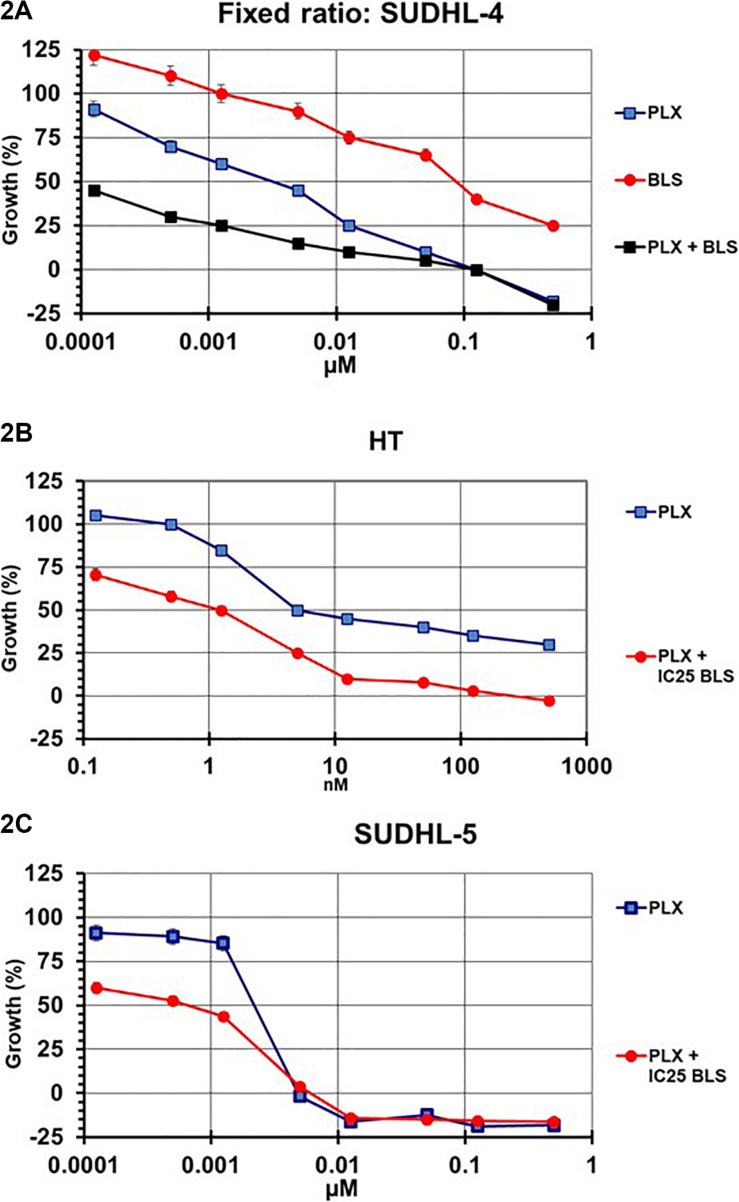
Example curves for the evaluation of the interaction between PLX and BLS in SUDHL-4 **(A)**, HT **(B)**, and SUDHL-5 **(C)** cells. SUDHL-4 cells were exposed to both drugs for 72 hr at a fixed molar ratio, based on the IC50 values of each drug **(A)**. SUDHL-5 and HT cells were exposed to a concentration gradient of PLX for 72 hr, in the presence of the IC25 of BLS **(B,C)**. The interaction between the two drugs was evaluated using the multidrug effect concept using the CalcuSyn program. Figures are of one representative experiment, performed in triplicate. The experiments were performed at least three times. Bars represent SEM, when not visible, they are within the size of the marker.

**FIGURE 3:**
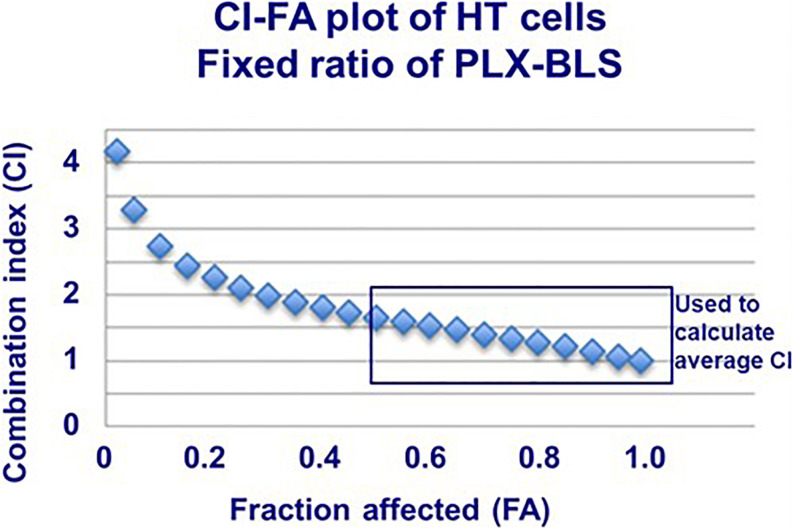
Example combination index (CI)-fraction affected (FA) curve for HT cells exposed to PLX and BLS in a fixed molar ratio based on the IC50. Drug exposure was 72 h. The SEM was within the size of the markers. This plot was fit by the program based on the actual values, showing antagonism/additivity at high FA values. The plot also indicates which part of the curve (actual CI values above FA > 0.5; usually the CI value around the FA of 0.5, 0.75, and 0.9) was used to calculate the average CI value of each experiment.

Initial experiments were designed according to the standard procedure of the median-effect program, simultaneous combination of the drugs at a fixed molar ratio based on the IC50 values. An example growth curve is shown in Figure 2A for SUDHL-4, which showed an additive/synergistic interaction. However, for all other cell lines the fixed ratio schedule resulted in either additivity or even antagonism (Figure 3 and Table 3). In a sequential administration of BLS pretreatment followed by PLX a similar effect, additivity or antagonism was observed (Supplementary Figure S1A and Table 3). Therefore we used a different approach in three cell lines in which we investigated whether PLX would increase the sensitivity to BLS or whether BLS would increase sensitivity to PLX. For this purpose we exposed cells to either the IC25 of PLX or BLS, respectively, in combination with the other drug in a concentration range (Figures 2B,C and Supplementary Figures S1B–E). The combination of BLS at its IC25 resulted in a synergism with PLX (Figures 2B,C and Supplementary Figure S2), but an IC25 of PLX with BLS appeared to be antagonistic/additive (Supplementary Figures S1D,E). Apparently, BLS shows a schedule-dependent potentiation of the effect of PLX (Table 3).

**TABLE 3:** Analysis of drug interaction between PLX and BLS in lymphoma cells using the median drug effect analysis.

Cell line	Fixed		Non-fixed	
	Simultaneous	BLS - > PLX	IC25 PLX	IC25 BLS
	CI	CI	CI	CI
HT	1.11 ± 0.14	1.35 ± 0.02	1.18 ± 0.31	0.40 ± 0.11
SUDHL-4	0.63 ± 0.15	0.95 ± 0.12	0.62 ± 0.16	0.66 ± 0.11
SUDHL-5	1.05 ± 0.23	1.26 ± 0.14	0.94 ± 0.11	0.72 ± 0.15
Jeko-1	1.22 ± 0.53			
Karpas-299	1.21 ± 0.28			
Hut-29	1.14 ± 0.23			

*Values are means ± SEM of three separate experiments, and are calculated from the average CI from FA values between 0.5 and 0.95 of each separate experiment. Drugs were combined at a fixed molar ratio based on the IC50 either simultaneously for 72 h or sequentially in which cells were exposed to BLS for 24 h followed by 48 h to both drugs at the same fixed ratio. Alternatively cells were treated with the drugs at a non-fixed ratio in which cells were exposed to the IC25 of one drug together with a concentration range of the other drug for 72 h.*

### Effect of the Drugs on Cell Death

**FIGURE 4:**
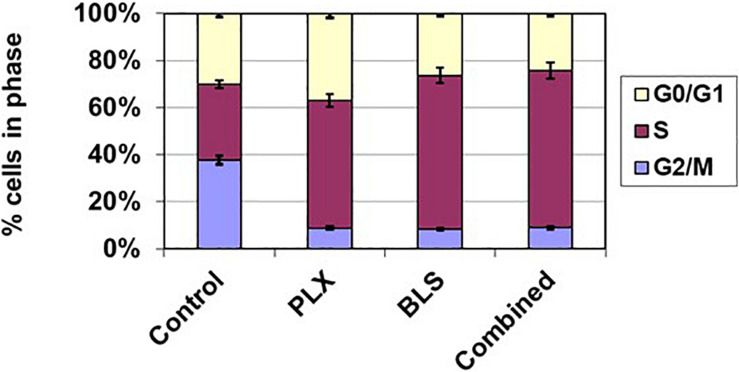
Cell cycle effects of BLS and PLX after 72 h exposure of HT cells to the drugs at their IC50 value. Data are from one representative experiment out of 3. Error bars represent SEM, when not visible, they are within the size of the marker.

**FIGURE 5:**
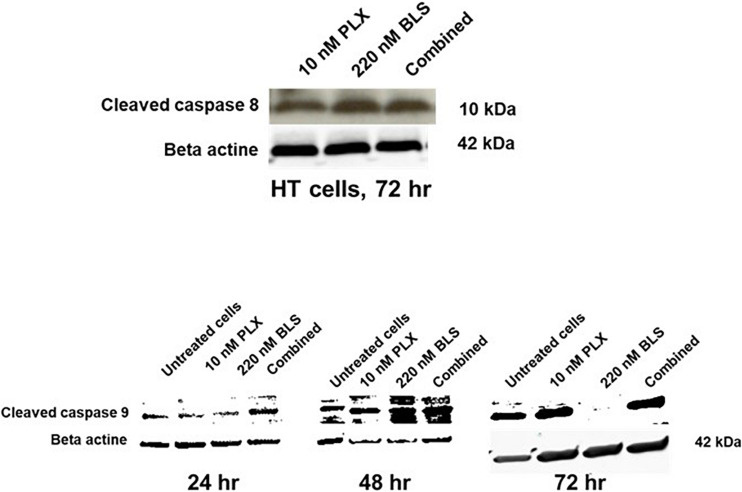
Induction of caspase 8 and caspase 9 cleavage in HT cells after exposure of the cells to their approximate IC50 (10 nM PLX, 220 nM BLS). The blots are a representative example out of three separate experiments.

Both drugs have been described as affecting the cell cycle and inducing cell kill, which might be enhanced by modulation via HDAC inhibition of DNA repair. Indeed both drugs increased accumulation of cells in the S-phase, which was more pronounced for BLS, while in the combination the effect was similar to that of BLS (Figure 4), FACS analysis also revealed an accumulation of cells in the sub-G1 phase an indicator of cell death. While untreated HT cells showed an accumulation of 3.5%, drugs at their IC50 showed a substantial increase to 13% for PLX, 21% for BLS and 23% for the combination. For SUDHL-4 cells these values were 5.0, 22.1, 15.5, and 28.1%, respectively. In order to determine which apoptotic pathway would be responsible for these effects we also investigated whether they would increase cleavage of either caspase 8 (the extrinsic pathway) or caspase 9 (the intrinsic pathway). No additive effect was found for caspase 8, but a time-dependent increase in cleavage of caspase 9 was found in HT cells (Figure 5). For SUDHL-4 similar data were observed (Supplementary Figure S3).

## Discussion

In this investigation we show that BLS can potentiate the effect of PLX when combined at a modulating concentration. However, most other combinations were additive. Both PLX and BLS have been tested earlier in various combinations, such as the combinations of PLX with gemcitabine in Non-Hodgkin Lymphoma models, in which sequential addition was five times more effective in inducing apoptosis compared with simultaneous exposure (Tonner et al., 2006). Unfortunately, the clinical studies did not show improvement (ClinicalTrials.gov: NCT00481871; Reungwetwattana et al., 2012). Interestingly, the combination of gemcitabine and BLS was also synergistic in three pancreatic cancer cell lines, in which a 1.5–3-fold increase was found for the pro-apoptotic activity of the combination as compared to BLS alone (Dovzhanskiy et al., 2012), while also in mouse models the combination was more effective than each drug alone (Chien et al., 2014). The latter studies were performed in pancreatic cancer cell lines and thus direct comparison with lymphoma cells is not possible. A multidrug combination of BLS with gemcitabine, azacitidine/busulfan/melphalan in refractory lymphoma was withdrawn (ClinicalTrials.gov: NCT02701673), and no further evaluation of the dual combination BLS and gemcitabine was performed yet.

The efficacy of BLS was comparable to earlier studies with lymphoma cell lines such as Diffuse Large B-Cell Lymphomas (DLBCL) cell lines with IC50 values in subnanomolar range (Tula-Sanchez et al., 2013), while also in solid malignancies IC50 values were in the nanomolar range, including pancreatic cancer cell lines (i.e., 100–600 nM) (Chien et al., 2014), urothelial carcinoma cell lines (Buckley et al., 2007) and in prostate cancer cell lines (Qain et al., 2008; Gravina et al., 2012). These data at least suggest that BLS may be a highly potent therapeutic agent for other lymphoma.

Earlier data also show a similarly high sensitivity of DLBCL for PLX (Marchi et al., 2010). The efficacy of PLX was shown to be related to the expression of RFC and DHFR (Kinahan et al., 2020). This is in line with the findings that PLX is an excellent substrate for the RFC (Figure 1; Wang et al., 2003; Visentin et al., 2014), but a poor substrate for PCFT and had a very low affinity for the folate receptors (Table 1). In addition, it was shown that PLX was one log more potent than methotrexate in suppressing cell proliferation (Tonner et al., 2006). We observed that LV can protect lymphoma cells against PLX, which means that timing of administration of LV is critical similar to that of post-treatment of LV after methotrexate (Wood and Wu, 2015). Earlier we demonstrated that LV can protect bone marrow cells more efficiently than tumor cells (both hematological and solid tumors) against edatrexate (Jolivet et al., 1994). Edatrexate was the prototype drug for development of pralatrexate (DeGraw et al., 1993; Wang et al., 2003). In fact, LV has been used to protect patients against toxic side effects (mucositis) of PLX (Foss et al., 2019).

Our data indicate that the interaction of BLS and PLX may be related to an increase in cell death, although both drugs were potent by themselves. For both BLS and PLX it was shown that they can induce cell death via the intrinsic and extrinsic apoptosis pathway (Hwang et al., 2009; Marchi et al., 2013), although our data indicate a higher contribution of the intrinsic pathway, because of the increased cleavage of caspase 9. This was not unexpected since intrinsic apoptosis was earlier shown to be the preferred apoptotic pathway in lymphoma cell lines (Cillessen et al., 2008). These data are thus in concordance with our current results and confirm that both BLS and PLX induce apoptosis, mostly likely via accumulation of cells in the S-phase. The accumulation of cells in the S-phase as was found in our cells was in line with earlier observations that PLX induces accumulation of DLBCL cells in the S-phase (Tonner et al., 2006). In earlier studies on HDAC combinations (Eckschlager et al., 2017; Verza et al., 2020), it was shown that a modulating concentration of the HDAC inhibitor is able to enhance the efficacy of DNA targeted drugs, such as the cross-linker cisplatin, possibly by down regulation of DNA repair enzymes by the HDAC inhibitor. This is in line with the interaction data described in the present study.

We did not proceed with testing PLX in *in vivo* mouse models, since earlier experiments by us and others clearly demonstrated that endogenous high folate levels in mouse blood and tissues (including tumor models) (Schmitz et al., 1994) protected these mice not only against systemic toxicity of methotrexate and antifolate based thymidylate synthase inhibitors but also reduced (or completely prevented) antitumor effect (Worzalla et al., 1998; Cao et al., 1999; Van der Wilt et al., 2001; Mauritz et al., 2008). Moreover, mice have a high level of thymidine in blood and tissues, which enables an additional protection. The latter can be bypassed by using models with a thymidine kinase deficiency, but no DLBCL and PTCL cell models with a thymidine kinase deficiency are available, making such studies less likely to be successful.

Altogether, these data show that a combination of PLX and BLS seems worthwhile to be explored in the treatment of B-cell lymphoma, since their activity is synergistic to additive in various model systems. However, these data also indicate that the combination should be evaluated carefully, since both drugs seem to induce this effect via similar apoptotic pathways, which may lead to increased toxicity to normal cells.

## Data Availability Statement

The raw data supporting the conclusion of this article will be made available by the authors, without undue reservation.

## Author Contributions

GJP, GR, and GJ: conceptualization. FPAvG and IK: experimentation. GJP and GJ: supervision. SAGM: cell lines and design of apoptosis. FPAvG and GJP: initial writing. All authors contributed to the article and approved the submitted version.

## Conflict of Interest

GJP and GJ received an educational grant from Spectrum Pharmaceuticals. GR was an employee of Spectrum Pharmaceuticals, Irvine, CA, United States. The remaining authors declare that the research was conducted in the absence of any commercial or financial relationships that could be construed as a potential conflict of interest.

## Supplementary Material

Supplementary Figure 1 Examples of various growth inhibition curves performed under different conditions. **(A)** HT cells, exposed to a range of BLS concentrations for 72 h and from 24 to 72 h to both drugs in a fixed ratio. **(B)** HT cells, exposed to BLS around its IC25 (100 nM) and a concentration range of PLX (non-fixed ratio) for 72 h. The triangles represent the theoretical curve for this combination; since this line is above the experimental combination line, this combination is synergistic. **(C)** SUDHL-4 cells exposed to the IC25 of BLS at a non-fixed ratio of a range of PLX concentrations for 72 h. **(D)** HT cells, exposed to PLX around its IC25 (5 nM) and a concentration range of BLS (non-fixed ratio) for 72 h. The triangles represent the theoretical curve for this combination; since this line is below the combination line, this combination is antagonistic. **(E)** SUDHL-5 cells exposed to PLX around its IC25 (4 nM) and a concentration range of BLS (non-fixed ratio) for 72 h. The triangles represent the theoretical curve for this combination; since this line overlaps the combination line, this combination is additive.

Supplementary Figure 2 Example of a CI-FA plot for HT cells exposed to an IC25 for BLS (100 nM) and a concentration range for PLX for 72 h. Using the CalcuSyn program the data were generated but CalcuSyn does not generate a fit curve for non-fixed ratios. Actual values are used for calculation of an average CI per experiment.

Supplementary Figure 3 Western blots of caspase 9 cleavage in SUDHL-4 cells exposed to 5.5 nM PLX, 70 nM BLS and their combination for 24, 48, and 72 h.
