# Supplementary material for: Schedule-Dependent Synergy Between the Histone Deacetylase Inhibitor Belinostat and the Dihydrofolate Reductase Inhibitor Pralatrexate in T-and B-cell Lymphoma Cells in vitro
Source: Front Cell Dev Biol. 2020 Oct 9;8:577215. doi: 10.3389/fcell.2020.577215 (PMC7581941; doi:10.3389/fcell.2020.577215)

Schedule-dependent synergy between the Histone Deacetylase Inhibitor Belinostat and the Dihydrofolate Reductase Inhibitor Pralatrexate in T-and B-cell Lymphoma Cells *in vitro*

Godefridus Johannes Peters, Frank H van Gemert, Ietje Kathmann, Guru Reddy, Saskia AGM Cillesen, Gerrit Jansen

Frontiers - Cell Dev. Biol. -Epigenomics and Epigenetics 2020; 8, 577215

DOI: 10.3389/fcell.2020.577215

Legends to supplementary figures:

Suppl. Figure 1:

Examples of various growth inhibition curves performed under different conditions.

A: HT cells, exposed to a range of BLS concentrations for 72 hr and from 24-72 hr to both drugs in a fixed ratio.

B: HT cells, exposed to BLS around its IC25 (100 nM) and a concentration range of PLX (non-fixed ratio) for 72 hr. The triangles represent the theoretical curve for this combination; since this line is above the experimental combination line, this combination is synergistic.

C: SUDHL-4 cells exposed to the IC25 of BLS at a non-fixed ratio of a range of PLX concentrations for 72 hr.

D: HT cells, exposed to PLX around its IC25 (5 nM) and a concentration range of BLS (non-fixed ratio) for 72 hr. The triangles represent the theoretical curve for this combination; since this line is below the combination line, this combination is antagonistic.

E: SUDHL-5 cells exposed to PLX around its IC25 (4 nM) and a concentration range of BLS (non-fixed ratio) for 72 hr. The triangles represent the theoretical curve for this combination; since this line overlaps the combination line, this combination is additive.

Sup Fig. 1

S 1A

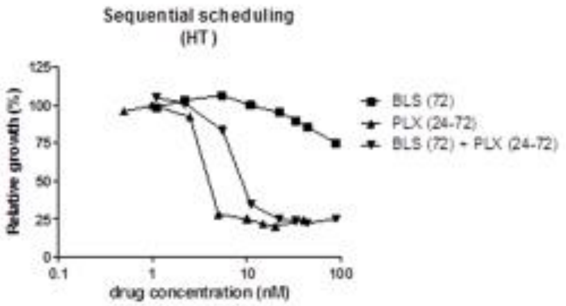

S 1B

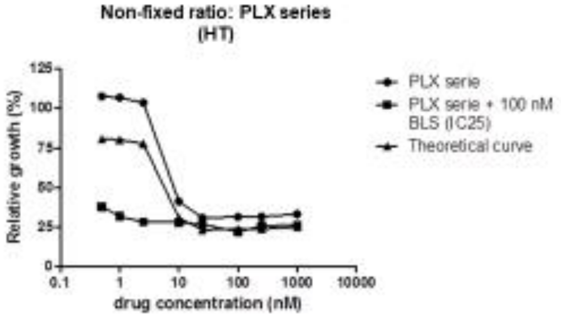

S 1C

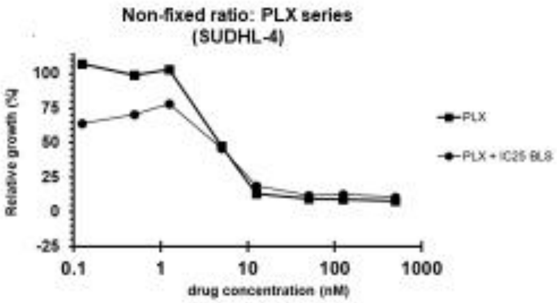

Sup Fig. 1

S 1D

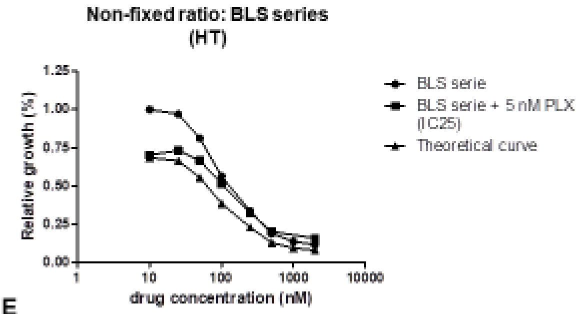

S 1E

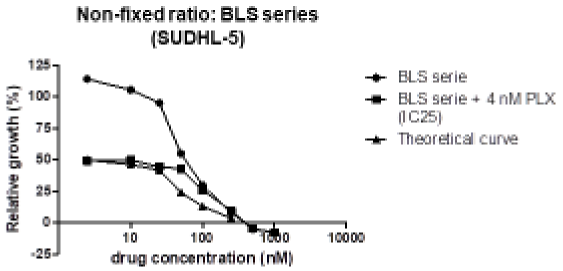

Sup. Fig. 2

Example of a CI-FA plot for HT cells exposed to an IC25 for BLS (100 nM) and a concentration range for PLX for 72 hr. Using the Calcusyn program the data were generated but Calcusyn does not generate a fit curve for non-fixed ratios. Actual values are used for calculation of an average CI per experiment.

Sup. Fig. 3:

Western blots of caspase 9 cleavage in SUDHL-4 cells exposed to 5.5 nM PLX, 70 nM BLS and their combination for 24, 48 and 72 hr.

Sup Fig. 2

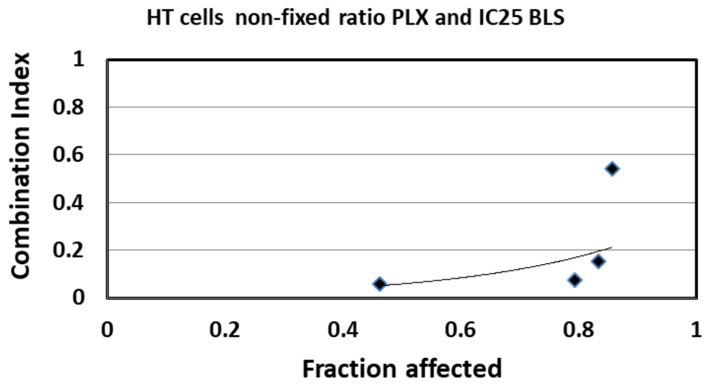

Sup Fig. 3

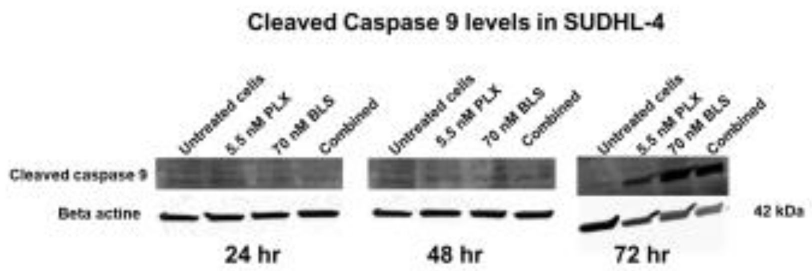

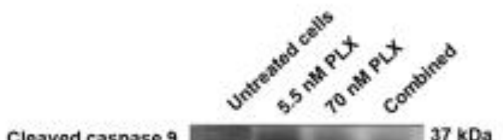

Supplement: Supplementary Figure 1 — Examples of various growth inhibition curves performed under different conditions. (A) HT cells, exposed to a range of BLS concentrations for 72 h and from 24 to 72 h to both drugs in a fixed ratio. (B) HT cells, exposed to BLS around its IC25 (100 nM) and a concentration range of PLX (non-fixed ratio) for 72 h. The triangles represent the theoretical curve for this combination; since this line is above the experimental combination line, this combination is synergistic. (C) SUDHL-4 cells exposed to the IC25 of BLS at a non-fixed ratio of a range of PLX concentrations for 72 h. (D) HT cells, exposed to PLX around its IC25 (5 nM) and a concentration range of BLS (non-fixed ratio) for 72 h. The triangles represent the theoretical curve for this combination; since this line is below the combination line, this combination is antagonistic. (E) SUDHL-5 cells exposed to PLX around its IC25 (4 nM) and a concentration range of BLS (non-fixed ratio) for 72 h. The triangles represent the theoretical curve for this combination; since this line overlaps the combination line, this combination is additive. [file Presentation_1.pdf]
